# The increasing role of structural proteomics in cyanobacteria

**DOI:** 10.1042/EBC20220095

**Published:** 2023-03-29

**Authors:** Jaspreet K. Sound, Jeddidiah Bellamy-Carter, Aneika C. Leney

**Affiliations:** School of Biosciences, University of Birmingham, Edgbaston, Birmingham, B15 2TT, U.K.

**Keywords:** Cyanobacteria, mass spectrometry, post translational modification, protein-protein interactions, proteomics

## Abstract

Cyanobacteria, also known as blue–green algae, are ubiquitous organisms on the planet. They contain tremendous protein machineries that are of interest to the biotechnology industry and beyond. Recently, the number of annotated cyanobacterial genomes has expanded, enabling structural studies on known gene-coded proteins to accelerate. This review focuses on the advances in mass spectrometry (MS) that have enabled structural proteomics studies to be performed on the proteins and protein complexes within cyanobacteria. The review also showcases examples whereby MS has revealed critical mechanistic information behind how these remarkable machines within cyanobacteria function.

## Introduction

Cyanobacteria, also known as blue–green algae, are among the oldest and most populous organisms on the planet. They have great potential in applications in biotechnology [1–3] including biofuel production [4], colorants [5,6], dietary supplements [7] and wastewater treatment [8,9]. Moreover, cyanobacteria can produce bioactive compounds with antiviral [10], anticancerous [11], antifungal [12] and antibacterial [13] activity making them an attractive research area. Understanding the genetic composition, the proteins cyanobacteria produce and how these function are important to fully exploit their biotechnological potential.

Until the early 2000s, obtaining cyanobacterial genome sequences was challenging due to the strong symbiotic relationship of cyanobacteria with other organisms and the strict requirement for axenic cultures for effective genome sequencing [14–16]. In addition, complications can arise since cyanobacteria can create disorder in their genome through horizontal gene transfer [17] when they adapt to new environments. Thus, even by 2017, the number of cyanobacterial genomic sequences was still relatively low compared with other bacterial phyla [18]. However, recent advances in metagenomics [18] that circumvent the need for axenic cultures are transitioning this research area and consequently the numbers of sequenced cyanobacterial genomes are now expanding rapidly. Alongside these developments, eDNA metabarcoding is providing a potentially highly effective approach for routine monitoring of cyanobacteria within cyanobacterial blooms [19,20].

Simultaneous to these developments, the field of cyanobacterial proteomics has also been expanding rapidly [21–24]. With developments in high resolution mass spectrometers that operate at high sensitivity, hundreds of proteins within cyanobacteria can effectively be screened to determine how cyanobacteria respond or adapt to environmental stimuli [25–27]. Moreover, now encoded protein sequences are available, structural biology experiments have begun to investigate the encoded proteins’ functional proteoform(s). This review will focus on these recent advances in structural proteomics and how the application of this technology has accelerated our in-depth understanding of the remarkable cellular processes within cyanobacteria.

Despite the focus of this review being proteomics, it is important that the integration of genomics and proteomics and the parallel advancement of both techniques continues. Indeed, due to the complexity of cyanobacterial genetic analysis, mistakes can occur within genome sequences that can subsequently be ‘corrected’ through proteomic analysis. For example, a proteogenomics study by Zhao and co-workers was able to correct 38 predicted gene-coding regions of the *Synechococcus sp*. PCC 7002 genome [28]. Single amino acid differences at the individual protein level have also been noted upon proteomic analysis. For example, the discrepancy between an arginine or alanine residue at position 21 in the β-subunit of allophycocyanin in *Arthrospira platensis* was confirmed using mass spectrometry (MS) to be alanine [29,30]. Indeed, through MS-based protein sequencing the predominant protein, phycocyanin, within the light harvesting complex of *Phormidium rubidium* was found to have 49 differences compared with its gene-derived sequence, which ultimately led to a more precise structure of its resulting complex [31]. Moreover, it is only with the knowledge of correct protein sequences within the cyanobacterial genome that we can perform structural studies to capture the details of how proteins and protein complexes function at the molecular level.

## Developments in structural mass spectrometry that have accelerated knowledge of the cyanobacterial proteome

Structural proteomics is the analysis of 3D protein structures with the aim to understand how proteins function on a genome-wide scale [32,33]. Traditionally, structural biology has focused on individual proteins or protein complexes with the aim to build mechanistic information about how they operate. Exciting bioinformatic developments in AlphaFold [34], mean protein structures can now largely be predicted from their primary sequence. Cryo-electron microscopy, in particular single particle cryo-electron microscopy, has overcome a major barrier in solving structures of large assemblies, being able to provide snapshots of complexes at atomic resolution [35,36]. However, alternative techniques are still required to fully capture protein dynamics and transient interactions, together with the large heterogeneity that is present within some functional complexes. Moreover, the structures of protein complexes can be challenging to predict, and we still rely on biophysical measurements to fully characterise their interactions. Additional complexity also occurs when proteins are dynamically modified with PTMs that themselves create heterogeneity within complexes and can drastically alter a protein’s function. It is to this end that structural MS can be advantageous.

**Figure 1:**
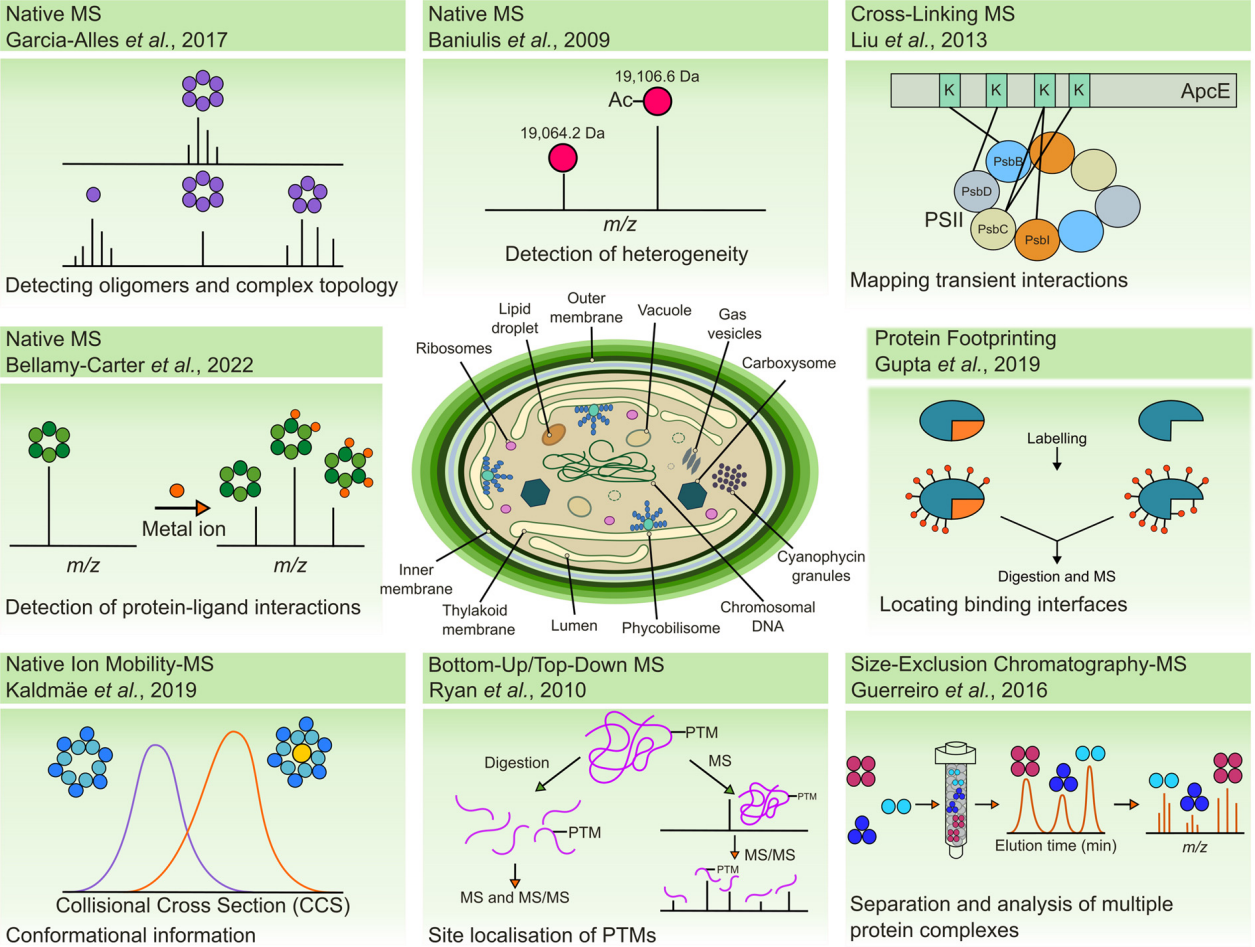
Structural information provided by MS The figure showcases how structural MS can provide information on oligomeric states and complex topology [56,57], protein–ligand or protein–metal interactions [58] and protein conformations [59] through native MS. PTMs can be determined through intact mass [60] combined with bottom-up and top-down approaches [61] with cross-linking MS [62] and protein footprinting [63] revealing information about transient interactions and protein binding interfaces.

MS alone, whereby a protein is infused directly into a mass spectrometer, has provided a wealth of information on protein structures within cyanobacteria (Figure 1). Through intact mass measurements, protein oligomeric states can be discerned, binding stoichiometry determined between proteins and ligands, and the extent and nature of any PTMs deciphered [37–39]. Tandem MS (MS/MS) on proteins or protein complexes provides additional information on protein stability and protein complex topology [40,41]. In combination with ion mobility spectrometry, the conformation of proteins and protein complexes can be revealed [42–44]. These aforementioned techniques encompass the basis of native MS whereby structural information is inferred by analyzing protein or protein complexes in as close conditions as possible to their cellular environment [38,45]. MS can also be used to map sites within protein interaction interfaces. Protein footprinting techniques, including most commonly used hydrogen deuterium exchange [46,47] and hydroxyl radical footprinting [48,49], can be used to probe in-solution differences in backbone interactions and side chain solvent accessibility, respectively, between different protein states. Within the gas-phase in the mass spectrometer, top-down fragmentation can additionally probe B factor or surface residues that reveal information on binding interactions and protein conformational states [50]. In addition, cross-linking provides a means to capture transient complexes or conformational states prior to MS analysis. By cross-linking amino acid side chains in close proximity and monitoring these sites of modification, structural constraints can be placed on proteins or protein complexes that enable models to be built or static structures to be re-evaluated [51–53]. Moreover, with on-going developments in online separation techniques (e.g. size exclusion chromatography) and data analysis tools, the wealth of information structural MS provides within a single study is being expanded beyond the single protein complex level towards deciphering the dynamics of hundreds of endogenous protein complexes [54]. Furthermore, since MS separates by mass-to-charge ratio, different protein proteoforms can be separated and the heterogeneity within complexes visualized [55], thus, showcasing how MS can be advantageous over other structural biology techniques. A summary of the types of information structural MS can provide are highlighted in Figure 1, pointing to exemplar studies of how each technique has been applied within cyanobacteria. In the next sections, we expand on the insight these techniques have brought to our understanding of PTMs and protein complex topologies within cyanobacteria, that together have built our knowledge on how key complexes within cyanobacteria function.

## Identifying post-translational modifications in cyanobacteria

**Figure 2:**
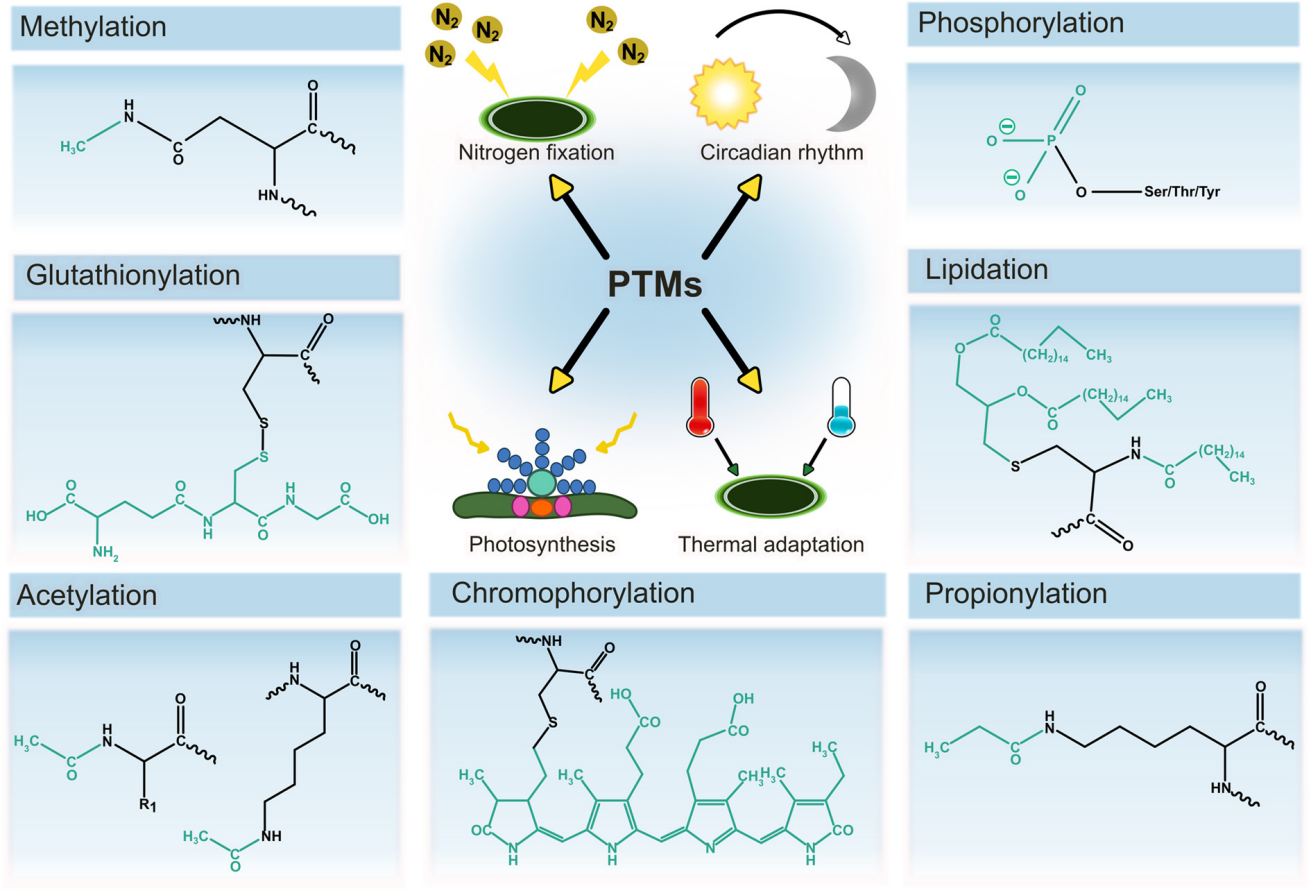
Selected protein PTMs that occur in cyanobacteria Seven key PTMs are highlighted including methylation, phosphorylation, chromophorylation, glutationylation, lipidation, propionylation and acetylation that collectively play roles in circadian rhythm, nitrogen fixation, photosynthesis and thermal adaptation. The protein side chains and PTM structures are shown in black and green, respectively.

MS excels in its ability to resolve minor mass differences on intact proteins and thus can distinguish many protein proteoforms. Through MS/MS experiments, the sites of PTMs can be localised and their abundance quantified [64–67]. These advantages of MS in PTM identification have meant that many PTMs, which play major roles in many physiological processes within cyanobacteria, have now been reported. Indeed, it is the PTM of phycobiliproteins with phycocyanobilins that provides cyanobacteria with their characteristic blue–green colour. These phycocyanobilin PTMs are essential to light harvesting and energy transfer within cyanobacteria’s photosynthetic machinery. Moreover, proteins that contain phycocyanobilins have been biotechnologically exploited for use as fluorescent probes [68–70], food colourants [71–73] and in cosmetics [74,75] due to their remarkable blue colour. However, PTMs are not confined to light harvesting complexes. Other essential processes within cyanobacteria that also heavily rely on PTMs include: nitrogen fixation [76,77], photosynthesis [78–84], thermal adaptation [85,86], chromatic adaptation [87–89], carbon/nitrogen control [90–94] and circadian rhythm [95–99] (Figure 2).

Overall, MS analysis of cyanobacterial proteins has identified several types of PTMs. For instance, proteomic studies determined 23 and 27 different types of PTM in *Synechococcus sp*. PCC 7002 [28] and *Nostoc sp*. PCC 7120 [100], respectively, using MS. These PTMs in cyanobacteria include, but are not limited to, phosphorylation [25,101–107], acetylation [60,108,109], glutathionylation [110], lipidation [111,112], propionylation [113,114], malonylation [115], methylation [82,83], succinylation [116,117] and glycosylation [118] as illustrated in Figure 2. The presence of these modifications can directly impact cellular processes. For example, the phosphorylation of Ser-49 of the P_II_ signal transduction protein is an important factor in signalling nitrogen deficiency [25,91,93], while the acetylation of K-190 of the protein PsbO is involved in the negative regulation of oxygen evolution in photosystem II [109]. Another notable example is on RexT, a redox-sensing transcriptional regulator, whereby MS helped locate Cys-41 as a critical cysteine residue that forms a vicinal disulphide bond in response to elevated hydrogen peroxide levels [119].

**Figure 3:**
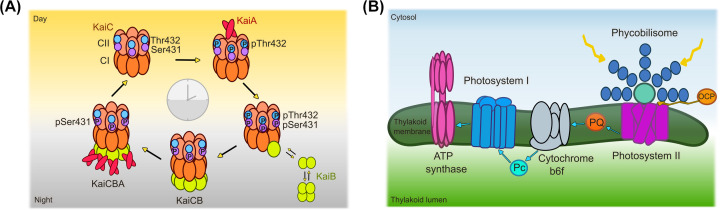
Schematic of the circadian clock and photosynthetic machinery within cyanobacteria (**A**) Schematic of KaiCBA protein complexes that form throughout the circadian cycle as informed by structural proteomics studies. (**B**) Cartoon images of the six major complexes: PBS, photosystem I and II, cytochrome b6f and ATP synthase are shown alongside plastoquinone (PQ), plastocyanin (Pc) and the OCP.

Another prominent example of PTMs in cyanobacteria is in controlling circadian rhythm. Within this, KaiC undergoes autophosphorylation, KaiA enhances autophosphorylation of KaiC, while KaiB antagonises it [96–98]. Moreover, phosphorylation of KaiC follows a four-step sequence whereby: (1) Thr-432 is phosphorylated, (2) Ser-431 is phosphorylated, (3) Thr-432 is dephosphorylated, and (4) Ser-431 is dephosphorylated, resulting in the non-phosphorylated form of KaiC which can subsequently restart the sequence [120,121] (Figure 3A). It is these oscillating levels of phosphorylation and dephosphorylation of KaiC that are essential for determining the phase of circadian rhythm [99,122,123]. The importance of PTMs in cyanobacteria is not limited to just the protein level. Cyanobactins, peptides produced as secondary metabolites, are heavily post-translationally modified with both prenylation [124–126] and geranylation [127] modifications reported. The findings of which have been reviewed in more detail elsewhere [128,129].

Overall, despite ancestral origin, the different types of PTMs found across cyanobacteria species are vast, with new modifications continuing to be discovered. As the diversity of MS methods to study PTMs evolves (Table 1), our ability to capture and monitor the function of these modifications will expand. We anticipate that the number of detected PTMs will also continue to increase with advancements in higher sensitivity instruments, the development of novel enrichment techniques for PTMs that occur sub-stoichiometrically, and enhanced bioinformatics tools that enable ‘open’ searching of MS/MS data without prior knowledge of the PTM of interest. However, these novel PTMs must be taken with caution, be carefully annotated to avoid mis-interpretation [130], and their presence confirmed *in vivo* through biological characterisation.

**Table 1:** MS methods used to identify protein PTMs in cyanobacteria

MS method	PTM information provided	Examples of PTMs observed using MS method
Native MS	Stoichiometry and nature of PTMs on native protein complexes	● Phosphorylation on KaiC and KaiCBA oligomers [131]
		● Bilin modification and methyl-asparagine on phycobiliprotein hexamers [30,132]
Top-down MS	Site-localisation and stoichiometry of PTMs on individual proteins	● N-terminal acetylation of PetC within the Cytochrome b_6f_ complex [61]
		● Lipidation of photosystem II assembly factors, Ycf48 [133] and Psb27 [111]
		● Bilin modification on cysteine residues within phycobiliproteins [134]
Bottom-up MS	Site-localisation of PTMs in a high-throughput approach	● Lysine methylation [135]
		● Phosphorylation on KaiC [136], ET-Tu [137] and PBS proteins [138]
		● Bilin modifications on phycobiliproteins [139]
		● Lysine propionylation (many sites detected in regulation of photosynthesis and metabolism) [113]
		● Glutathionylation of peroxiredoxin and 3-phosphoglycerate dehydrogenase [110]
		● C-terminal processing of D1 in photosystem II [140]

## Identifying novel protein–protein interactions

MS studies on cyanobacteria have led to the identification of novel protein complexes that are dependent upon the cyanobacteria’s cellular context. These findings have often been a result of looking at intact protein complexes rather than individual proteins. Indeed, if a novel protein is detected within a macromolecular complex of known function, this on its own provides functional insight into the role of the newly identified protein, which may alter in response to cellular stimuli. Moreover, a study by Guerreiro et al. noted that when looking at global protein levels, the fluctuation in proteins in response to light was not very pronounced [141]. However, when native protein complex fractionation (size exclusion chromatography) was combined with high resolution proteomics, large complex assemblies including ribosomal and photosynthetic complexes were observed to change in response to light [57]. Within these data, more component variety was observed within photosynthetic complexes in the light phase, a finding that would be undetectable at the individual protein analysis level. In another light-dependent study, through pull-down experiments followed by MS analysis, a collection of proteins that are directly or indirectly associated with the vesicle-inducing protein in plastid 1 (Vipp1) were identified only after exposure to light suggesting that Vipp1 may be involved in protein assembly [142]. In more recent studies, Xu et al*.* used a combination of size-exclusion chromatography, ion exchange chromatography and sucrose density gradient centrifugation followed by MS, collectively termed Co-Frac-MS, to map the protein interactome within *Synechocystis sp*. PCC 6803 revealing new insights into photosynthesis, cell mobility and lipid metabolism [143]. With additional developments in native polyacrylamide gel electrophoresis, these MS studies can now be expanded to the analysis of membrane complexes that are more challenging to analyse by conventional separation techniques [144].

On the structural level, the ability of native MS to monitor oligomeric states of proteins and determine protein complex topology has been utilised to reveal new insight within several protein complexes within cyanobacteria. Hackenberg et al. used native MS to show that the cystathionine β-synthase (CBS)-chloroplast protein (CP12) fusion protein, as a single entity, can form a hexameric structure that has suggested roles in redox regulation [145]. Eisenberg et al. were able to monitor how the oligomeric state of phycocyanin increases with increasing concentration, which might indicate how light harvesting systems adapt to a range of environmental conditions [146]. In addition, elegant studies by Clarke and co-workers have revealed the complex topology of two different Clp proteases, ClpXP1/P2 [147] and ClpP3/R [148], that operate to drive protein substrate unfolding prior to proteolytic degradation.

Other work of note where determining the oligomeric statuses’ of proteins has been advantageous is centred around the carboxysome; the compartments within cyanobacteria that are responsible for fixing carbon from inorganic substances [149]. The assembly of the carboxysome shell follows a complex series of events. Garcia-Alles et al. showed CcmK can assemble into hexameric structures [150] with some isoforms forming higher order structures that are dynamic in nature [151]. These CcmK assemblies then further aggregate to form the faces of the carboxysome. Inside the carboxysome reside the enzymes ribulose 1,5-bisphosphate carboxylase/oxygenase (RuBisCO) and carbonic anhydrase that together act to fix carbon dioxide. Native MS in combination with size exclusion chromatography coupled to right-angled light scattering showed that the small domains within CcmM, a protein involved in RuBisCO recruitment to the β-carboxysome, bind independently of the RbcS subunit, a small subunit of RuBisCO, suggesting it locates within an extended electronegative pocket between the RbcL dimers of RuBisCO, contrary to previously predicted interactions [152]. In another study on RuBisCO, its metabolic repair mechanism by the AAA+ chaperone RuBisCO activase (Rca) was investigated [153]. Native MS confirmed the stable hexameric state of the engineered Rca complex which, using a combination of hydrogen-deuterium exchange MS, cross-linking MS and cryo-electron microscopy, was shown to have conformational effects on RuBisCO’s catalytic site upon interaction [153].

## Providing deeper insight into macromolecular machine function

With the increasing developments in structural MS techniques, not only are new proteins and their interaction partners being discovered, but detailed mechanistic information of how key complexes function within cyanobacteria are becoming apparent. Two prominent examples of protein machineries that are noteworthy of further discussion are the circadian clock and photosynthesis.

As mentioned previously, phosphorylation is key in controlling circadian rhythm. In addition to mapping sites of phosphorylation on KaiC [120,121], native MS studies have taken strides in revealing how phosphorylation can alter Kai’s multi-component complexes (Figure 3A). Initial native MS studies into the circadian system showed that KaiB can form monomers, dimers, and tetramers, whereby KaiB binds as a monomer to KaiC in a cooperative fashion to form a KaiC_6_B_6_ complex [154]. Following this, native MS was used to prepare well-defined stoichiometric assembles of KaiCB and KaiCBA (specifically KaiC_6_B_6_ and KaiC_6_B_6_A_12_) that enabled their structural characterisation by single particle cryo-electron microscopy together with hydrogen-deuterium exchange MS and cross-linking MS [155]. Since then, native MS has further shown that autophosphorylation of hexameric KaiC can promote its binding to dimeric KaiA [156]. These data together provided a structural basis to understand complex assembly within the oscillating clock.

Another area where structural MS is making strides forwards is in our understanding of photosynthesis [157]. There are six major complexes that combine to aid photosynthesis within cyanobacteria: the phycobilisome (PBS), photosystem I and II, cytochrome b_6_f, NAD(P)H quinone oxidoreductase complex, and ATP synthase (Figure 3B). The PBS is a large light harvesting complex consisting of phycocyanin rods, connected by linker proteins, and an allophycocyanin core. Early work using gentle *in vivo* cross-linking followed by MS analysis was able to capture weak interactions within the large scale organisation of the PBS and photosystems, showing both photosystem I and II could interact with the PBS [62]. Later cross-linking work was able to predict potential docking interactions between the phycocyanin rods and the allophycocyanin core [158], and suggest a side-view crossover configuration of the two basal cylinders within the PBS core [159]. Furthermore, other studies have shown ferredoxin-NADP^+^ oxidoreductase, an enzyme involved in electron transport, and non-bleaching protein A, a proteolytic adapter protein, bind phycocyanin to fine-tune energy transfer [160] and PBS degradation [161], respectively. This MS work together combined with the most recent cryo-electron microscopy structures of the PBS [162,163] are aiding significantly in our understanding of how these light harvesting complexes function so efficiently.

When light levels are too high during photosynthesis, protective mechanisms within cyanobacteria must be in place to prevent photodamage. Orange carotenoid protein (OCP) has known photoprotective capabilities and binds to the PBS when light levels are too high [164–166]. Native MS revealed that OCP dimerises to different extents between its active and inactive forms [167]. The conformational differences between the two states have also been probed by footprinting MS [63,168–171]. Using cross-linking MS studies, the N-terminal domain of the active OCP was further found to bury into the PBS, changing the conformation of the allophycocyanin core, resulting in decoupling of light transfer from the PBS towards photosystem II [172,173]. This leaves the C-terminal domain of OCP exposed for binding to the dimeric fluorescence recovery protein that then converts OCP back to its inactive state [169,174]. Like the PBS, photosystem II also needs to be protected from photo-induced damage. Photosystem II is a multi-component protein complex, predominantly composed of reaction centre proteins (D1 and D2), cytochrome b_559_, and the chlorophyll-containing proteins (CP43 and CP47), that is responsible for water splitting, oxygen evolution and plastoquinone reduction. Two proteins, Psb27 and Psb28, are important in the successful repair of photosystem II [112,175]. A combination of cross-linking MS and protein footprinting MS studies have shown that Psb27 binds CP43 leading to the recruitment other proteins [176–178]. In contrast, Psb28 was found to bind to the CP43-less assembly intermediate known as RC47 [179]. Using an isotope encoded chemical cross-linker and MS, Psb28 was further found to bind to the cytosolic side of cytochrome b_559_, acting to protect the photosystem II subcomplexes until the photosystem II is ready to function [180].

Together, these MS studies have revealed insight into the PBS, photosystem I and photosystem II within the photosynthetic machinery of cyanobacteria. However, these structural MS studies are only the beginning with the developments in Alphafold 2 now providing more insight into even the intrinsically disordered regions within the PBS that can be further refined using structural MS [181]. We foresee this combined knowledge will accelerate our understanding on how all the photosynthetic complexes within cyanobacteria orchestrate to form the optimal functioning photosynthetic machinery.

## Conclusion

Structural proteomics can provide a wealth of information on how proteins function. Within cyanobacteria, structural MS has played a pivotal role in deciphering protein post-translational states, determining protein interaction partners, and revealing mechanistic details behind how proteins function. In this review, we have showcased examples of how structural MS has provided information on circadian rhythm, carbon fixation and photosynthesis. However, we envisage that many macromolecular complexes within cyanobacteria are yet to benefit from structural MS studies, the knowledge of which will significantly advance our understanding of how cyanobacteria function and produce their remarkably efficient protein machines.

## Summary

- Cyanobacteria are bursting with biotechnological potential.
- Advances in structural mass spectrometry are providing great insight into protein proteoforms and their interaction partners that together provide insight into how these function within cyanobacteria.
- This review highlights examples of where structural mass spectrometry has advanced our knowledge of important molecular mechanisms within cyanobacteria.

## Abbreviations

ATP: adenosine triphosphate
eDNA: environmental DNA
LC: liquid chromatography
MS: mass spectrometry
MS/MS: tandem mass spectrometry
NADPH: nicotinamide adenine dinucleotide phosphate
OCP: orange carotenoid protein
PTM: post-translational modification
RuBisCO: ribulose 1,5-bisphosphate carboxylase/oxygenase
